# Benefits from the correction of vitamin D deficiency in patients with pulmonary hypertension

**Published:** 2016

**Authors:** Ahmad Mirdamadi, Pouya Moshkdar

**Affiliations:** 1 Department of Cardiology, Islamic Azad University, Najafabad Branch, Isfahan, Iran.; 2 Islamic Azad University, Najafabad Branch, Isfahan, Iran.

**Keywords:** Vitamin D deficiency, pulmonary hypertension, Replacement therapy

## Abstract

**Background:**

Vitamin D (Vit D) is linked to various conditions including musculoskeletal, metabolic and cardiopulmonary diseases. However, it is not clear whether correction of vit D deficiency exerts any beneficial effect in patients with pulmonary hypertension.

**Methods:**

This study was a prospective uncontrolled longitudinal study. Patients with pulmonary hypertension and vit D deficiency were enrolled into this study. All patients in addition to standard treatment for pulmonary hypertension received cholecalciferol at a dose of 50,000 IU weekly plus calcicare (at a dose of 200 mg magnesium + 8 mg zinc + 400 IU vit D) daily for 3 months. Serum level of 25-hydroxy vit D, serum level of pro-brain natriuretic peptide, six minute walk test (6MWT), peak and mean pulmonary artery pressure, right ventricular size and function, ejection fraction (EF) and New York Heart Association (NYHA) functional class were measured at baseline and after 3 months of treatment.

**Results:**

Twenty-two patients with pulmonary hypertension and vit D deficiency were enrolled into the study. At endpoint, the serum vit D level increased significantly to 54.8 ng/ml, the mean of baseline distance of 6MWT increased significantly to 81.6 m and the RV size significantly improved. The mean pulmonary artery pressure also improved after the intervention, but their changes did not reach to statistically significant levels.

**Conclusion:**

Vit D replacement therapy in patients with pulmonary arterial hypertension and vit D deficiency results in significant improvement of right ventricular size and 6 MWT. Moreover, mean pulmonary artery pressure improves nonsignificantly. This issue requires further studies with long-term follow-up period.

Pulmonary arterial hypertension (PAH) is a progressive disorder characterized by mean pulmonary artery pressure ≥ 25 mm Hg, leading to right heart failure and reduced cardiac output with consequent mortality (1). Based on the 5th World Symposium clinical classification, held in Nice, France, in 2013, five groups of disorders that cause pulmonary hypertension were identified: 

Pulmonary arterial hypertension (Group 1); 

Pulmonary hypertension due to left heart disease (Group 2); 

Pulmonary hypertension due to chronic lung disease and/or hypoxia (Group 3);

Chronic thromboembolic pulmonary hypertension (Group 4); 

Pulmonary hypertension due to unclear multifactorial mechanisms (Group5) (2).

The factors that have been shown to predict survival in patients with pulmonary hypertension include baseline NYHA functional classification (3), history of right heart failure, baseline six minute walk distance (4), and baseline hemodynamics (5, 6). 

On the other hand, vit D deficiency is a major worldwide health problem that its prevalence in the general population is approximately 30% to 50% (7). Studies among Iranian population indicate a high prevalence of vit D deficiency and insufficiency in more than 50% of general population (8). The primary role of vit D on bone metabolism has extended to various clinical and pathophysiological conditions from osteomalacia to several immunologic, metabolic, cancer, infectious and cardiovascular diseases (9-11). Vit D deficiency stimulates the renin-angiotensin-aldosterone system (RASS) and leads to hypertension and left heart hypertrophy (12). 

Additionally, various studies have shown the relationship between vit D deficiency and thromboembolism (13, 14). Vit D affects the vascular endothelial growth factor (VEGF) and endothelin expression and vascular smooth muscle cell proliferation and can predispose to endothelial dysfunction (15-17).

Since the main pathogenic mechanisms of pulmonary hypertension are endothelial and smooth muscle cell proliferation, vasoconstriction, thrombosis and inflammation, it can be proposed that vit D may play a role in this pathophysiological vit D deficiency may exert inappropriate effect on the course of pulmonary hypertension (18-20). 

Some studies have shown that vit D therapy improve vascular health markers such as endothelial function, but it is not well-known whether vit D replacement therapy in patients with vit D deficiency and pulmonary hypertension improves pulmonary function. 

We, therefore, conducted this study to evaluate the effect of vit D replacement therapy on the cardiovascular parameter in patients with pulmonary hypertension who had also vit D deficiency.

## Methods

**Study Design and Participants: **This study was a prospective uncontrolled before - after clinical trial (IRCT2013091713828N3), designed to evaluate the effects of vit D replacement therapy in patients with pulmonary artery hypertension and vit D deficiency. This trial was conducted in a referral university hospital in Isfahan (Iran’s third largest city, located in the center of Iran), Iran. The Medical Ethics Committee of Isfahan University of Medical Sciences approved the study design, protocols, and informed consent was taken from all enrolled patients (the ethical code was 492030).

A total of 54 patients with pulmonary hypertension (mostly type1) were considered to be eligible for the study. Finally, 28 patients were enrolled. Twenty-two patients with pulmonary hypertension had vit D deficiency in this study. We included patients who were 20 to 65 years of age diagnosed with pulmonary hypertension (mean pulmonary artery pressure was greater than 25 by catheterization) and vit D deficiency or insufficiency (serum 25-hydroxy vit D level less than 30 ng / ml). 

All patients were clinically and hemodynamically stable. They were undergoing combination therapy for at least 1 year and we did not up titrate the pulmonary vasodilators. We excluded patients who were already taking vitamin D supplements or not contented to enter the trial.

The sample size was calculated on the assumptions to detect a difference of 15 (mmHg) in mean pulmonary artery pressure, with α = 0.05 and power= 80%. We considered 10% attrition rate. 

**Intervention and Measurements: **At entry into the study, sex, age, and type of pulmonary hypertension (based on the 5th World Symposium clinical classification, held in Nice, France, in 2013 organized regarding etiology) (2) of all participants were recorded. Also, all participants were examined for following measurements:

**Biochemical assessment**: Serum level of 25-hydroxy vit D was measured by chemiluminescent immunoassay (DiaSorin LIAISON, Inc.). Patients with serum 25-hydroxy vitamin D level less than 30 ng / mL were enrolled in the study. In addition, serum level of pro-brain natriuretic peptide (Pro-BNP) was analyzed by commercially available immunoassay (Elecsys, Roche diagnostics, Indiana) 

**6MWT analysis **(21, 22): The 6MWT (six minute walk test)was measured to assess the functional exercise capacity of patients. This test measures the distance (on a meter scale) that an individual can quickly walk in a period of 6 minutes on a flat, hard, indoor surface. The minimum clinically important change for the six-minute walk is 30 meters. Before the test starts, the participants sat at rest for at least 10 minutes. During this time, oxygen saturation (O2 Sat) was measured by pulse oximetry. At the end of the test, O2 Sat was measured again. 

**Echocardiography** **:** Patients underwent transthoracic echocardiography according to the American Society of Echocardiography (23) before and after trial for the assessment of the peak and mean pulmonary artery pressure, the right ventricular size and function and the ejection fraction. All echocardiograms were performed by an experienced fellowship of echocardiography.

**Functional class: **Patients were classified according to New York Heart Association (NYHA) functional class (class I =without limitation of physical activity, class II =slight limitation of physical activity, class III= marked limitation of physical activity, class IV = inability to carry on any physical activity without discomfort) (24).

All patients who had the inclusion criteria to be enrolled in the study received soft gelatin capsule of cholecalciferol (vit D3) (Zahravi Pharmaceutical company, Iran) at a dose of 50,000 IU weekly and calcicare table (Vitane Pharmaceuticals, Germany) at a dose of 200 mg magnesium + 8 mg zinc + 400 IU vit D daily, for 3 months. After 3 months of intervention, the serum level of 25-hydroxy vitamin D was checked and for those patients with serum level of vit D that remained less than 30 ng / mL, the second period of treatment was repeated. After intervention, all patients underwent biochemical, 6MWT, echocardiography and function class assessments in the same way of the first measurements at entry into the study. 

**Statistical Analysis: **The bio-statistical evaluation was carried out using SPSS Version 20 (release 2012 SPSS Inc., Chicago, IL) for windows. Qualitative data shown as number and relative frequencies and quantitative data presented as mean and standard deviation. The differences of quantitative, normally distributed data (diagnosed by the Kolmogorov Smirnov test) before and after intervention were assessed by paired t-test. For the data or qualitative data that were not normally distributed the Wilcoxon and McNemar statistics were used. All tests were two-sided and statistical significance was considered at the 0.05 probability level. 

## Results

Out of the total 54 patients with pulmonary hypertension (mostly group 1, based on clinical classification of pulmonary hypertension) who were deemed to be eligible only 28 patients were enrolled into the study. After evaluation of vit D level, a total of 22 patients showed to be suffering from vit D deficiency (approximately 80% prevalence of vit D deficiency in our patients). One patient died in the course of trial due to severe underlying disease and 21 patients completed the trial. All patients were on a standard target pulmonary hypertension treatment by endothelin-receptor-blocker (Bosentan) and phosphodiesterase inhibitor (sildenafil or tadalafil). Baseline characteristics of patients are described in table 1. 

**Table 1:** Baseline characteristics of the patient (n=22)

**Characteristics**	**Number (%)**
Age mean±SD	42.6(12.6)
Sex	
Male Female	4(18) 18(82)
Type of pulmonary hypertension	
IPAH Eisenmenger syndrome CTEPH Associated PH Scleroderma	9(43) 4(17) 5(22) 3(14) 1(4)
Drug history	
Spironolactone Bosentan Sildenafil Tadalafil Warfarin Digoxin	15(68) 22(100) 16(73) 6(27) 18(82) 2(9)

Data are presented as mean±SD or numbers (%)

**Fig 1:**
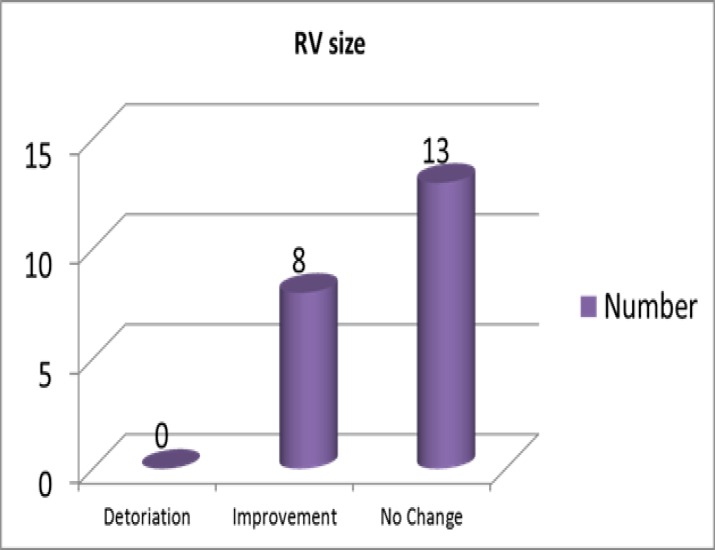
Effect of 3 months vitamin D replacement therapy on right ventricular size in patients with pulmonary artery hypertension and vitamin D deficiency

SD: Standard deviation; IPAH**:**Idiopathic Pulmonary arterial hypertension; CTEPH: Chronic Thromboembolic Pulmonary Hypertension;

After the intervention, serum vit D level improved significantly by 54.8 ng/ml and reached to > 30 ng/ml in all patients (table 2). RV size analysis showed that after vit D replacement therapy, RV size significantly improved (P=0.01) (figure 1). 

Analysis of the 6MWT showed that the mean of baseline distance statistically increased by 81.6 m after the intervention (P<0.001) (table 2) but Pre-6MWT and post-6MWT, O2 saturation did not statistically differ before vit D supplementation compared with after intervention (P=0.81, P=0.29, respectively). 

**Fig 2:**
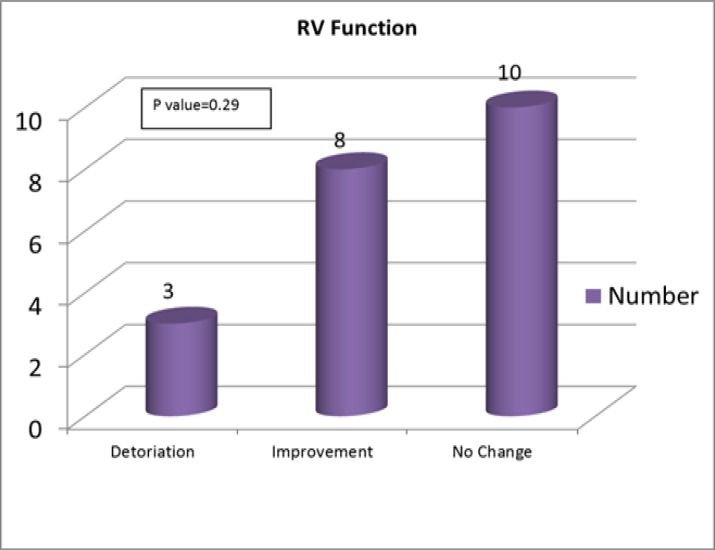
Effect of 3 months vitamin D replacement therapy on right ventricular function in patients with pulmonary artery hypertension and vitamin D deficiency

Besides, as presented in table 2, mean-PAP improved after the intervention but their changes were not statistically significant (p>0.05) (figure 2). Serum pro-BNP level and NYHA functional class do not significantly change (P=0.66, P=0.22, respectively) (table 2).

**Table 2:** Comparison of parameters before and after receiving vitamin D supplements in patients with pulmonary artery hypertension and vitamin D deficiency

**Parameters**	**Before** **N=22**	**After** **N=21**	**P value**
serum vitamin D level (ng/ml)	14.4(9.2)	69.2(31)	<0.001*
serum Pro-BNP level (ng/ml)	436(47.6)	486(60.7)	0.66*
6MWT(meter)	259.9(124.3)	330.5(139.2)	<0.001*
Pre6MWTO2Sat (%)	90.7(8.9)	90.5(8.4)	0.81*
Post6MWT O2Sat (%)	87.7(8.9)	88.9(9.4)	0.29*
EF (%)	53.8(3.9)	55.2(4)	0.14*
Peak-PAP(mmHg)	93.4(20.4)	92.41(24.8)	0.81*
Mean-PAP(mmHg)	79.75(24.92)	69.5(23.04)	0.07*
Functional Class (number (%))			0.22**
I II III IV	0 9(41%) 11(50%) 2(9%)	0 12(55%) 9(41%) 0	
RV size (number (%))			0.01**
Preserved Mild enlargement Moderate enlargement Severe enlargement	1(4.5%) 2(9%) 5(22.7%) 14(63.8%)	2(9%) 4(19.1%) 5(23.8%) 10(48.1%)	
RV Function (number (%))			0.29**
Normal Mild dysfunction Moderate dysfunction Severe dysfunction	4(18.3%) 6(27.3%) 8(36.1%) 4(18.3%)	6(28.5%) 6(28.5%) 6(28.5%) 3(14.5%)	

Data are presented as mean (Standard deviation) and number (%); 6MWT: Six Minute Walk Test; O2Sat: Oxygen Saturation; EF: Ejection Fraction; PAP: Pulmonary Artery Pressure; Function class I = without limitation of physical activity, class II = slight limitation of physical activity, class III= marked limitation of physical activity, class IV = inability to carry on any physical activity without discomfort; RV: Right Ventricle;

* Paired t-test;

* * Wilcoxon test;

## Discussion

Pulmonary hypertension is a life-threatening disease characterized by a progressive increase in pulmonary vascular resistance leading to heart failure and reduced cardiac output in which these conditions will lead to death (25). There are various methods of management to control this condition, but the patient may be affected by other complications, such as vit D deficiency in the course of the disease (26, 27).

Vit D seems to be related to blood pressure control via various pathways and is inversely correlated to serum renin activity (11). Vit D can suppress the renin activity, may be due to increased intracellular calcium levels (13). Vitamin D replacement therapy in deficient patients significantly improved brachial artery flow-mediated dilatation (27). 

Multiple lines of evidence suggest a link between vit D and cardiovascular disease, including experimental studies identifying vit D receptors in vascular smooth muscle cells and endothelial cells and possibly cardiac tissue (28).

The present study addressed the potential effect of vit D replacement on the improvement of cardiovascular function in patients with pulmonary hypertension and vit D deficiency. In the current setting, we found that vit D replacement therapy can correct vit D deficiency in most patients even with a short period of 3 months treatment. The increase in vit D level is accompanied by improvement of six-minute walk test (6MWT) as a well-known prognostic factor in these patients. In agreement with our finding, Boxer RS et al. demonstrated that longer six minute walk distance was correlated with higher 25-hydroxy vit D level (29). In contrast, other studies report that vit D supplement has no significant clinical effect on the six-minute walk distance in heart failure patients (30, 31). Perhaps the discrepancies in these studies are due to differences in the patient characteristics such as underlying disease.

Another finding of our study is the improvement in right ventricle size. Witte KK et al. concordant to our findings, showed right ventricle volumes were reduced by vit D supplement in heart failure patients (31).

 Though other studies failed to demonstrate any significant effect of vit D replacement therapy on right ventricular size (28). 

In our study, NYHA functional class was not affected by vit D replacement therapy. Similar to our finding, Zittermann A et al. revealed that there was no clear association between NYHA-functional class and 25 (OH) D levels (32).

We found no clear changes in ejection fraction after vit D replacement. This is while Witte KK et al. report that EF increased by 5.3% with vit D supplement therapy (31). We found a considerable decrease in mean pulmonary artery pressure, but this improvement is not statistically significant and may be due to the small sample size. Even so, another study revealed a strong correlation between pulmonary artery pressure and serum vit D level (19). Although serum pro-BNP level did not illustrate a significant change after intervention in our study, some studies showed a nonlinear regression analysis in 25-hydroxy vit D which is inversely correlated with Pro-BNP level (31, 32). A small sample size and differences in the patient characteristics may explain these discrepancies. According to the main limitation of our study, due to not having a control group in the study, we must interpret with caution that such improvement was exactly caused by vit D therapy.

 We did not allocate control group because of ethics, in addition to the small number of patients with type 1 of pulmonary hypertension in the community. Other limitations of our study included short duration of follow-up, lack of data regarding extra cardiac variables like muscle strength, presence of skeletal pain or musculoskeletal diseases which may have been corrected after normalization of serum vit D and resulted to walking improvement. Evaluation of muscle strength and musculoskeletal condition is a great recommendation that we will consider in future studies. 

Finally, our study showed that according to the high prevalence of vit D deficiency in general population and among patients with pulmonary arterial hypertension, attention to screening and treatment of vit D deficiency in these patients is important. Moreover, vit D replacement therapy is not expensive and has no significant adverse effects, on the contrary it has some advantages. In conclusion, the results of this study revealed that treatment of vit D deficiency in patients with pulmonary arterial hypertension has a significant effect on right ventricular size and 6MWT improvement. And mean pulmonary artery pressure demonstrated some improvements after replacement therapy, although it was not statistically significant.

## Notes

**Funding:** This project was funded by the authors.
